# Mixed response and mechanisms of resistance to larotrectinib in metastatic carcinoma ex pleomorphic adenoma of the parotid harboring an *NTRK2* fusion: A case report

**DOI:** 10.1097/MD.0000000000024463

**Published:** 2021-01-29

**Authors:** Magdalena Pircher, Hans Rudolf Briner, Marco Bonomo, Milo Horcic, Ulf Petrausch, Daniel Helbling, Thomas Winder

**Affiliations:** a Onkozentrum Zürich; b ORL-Zentrum Klinik Hirslanden, Zurich; c Clinica Luganese Moncucco, Lugano; d Institut für Histologische und Zytologische Diagnostik AG, Aarau; e Swiss Tumor Molecular Institute, Zurich, Switzerland.

**Keywords:** carcinoma ex pleomorphic adenoma, larotrectinib, *NTRK2* fusion

## Abstract

**Introduction:**

Standardized systemic treatment options are lacking for carcinoma ex pleomorphic adenoma, which is a rare and aggressive tumor primarily found in salivary glands.

Here we report the case of a 63-year-old male with carcinoma ex pleomorphic adenoma of the left parotid and parapharyngeal space harboring a neurotrophic receptor tyrosine kinase (*NTRK) 2* fusion who was treated with a small molecule inhibitor that targets the tropomyosin receptor kinase (TRK) proteins. To the best of our knowledge, no similar case has been described in the literature so far.

**Patient concerns:**

After multiple surgical resections and radiotherapy for localized cancer disease over several years, our patient again developed an increasing swelling and pain around the left ear and numbness of the left half of the face.

**Diagnosis:**

Magnetic resonance imaging and positron emission tomography/computed tomography scans showed tumor recurrence in the left parotid, below the left ear, and in the parapharyngeal space, as well as metastases of the lungs and cervical lymph nodes. As data on the efficacy of systemic therapies for inoperable carcinoma ex pleomorphic adenoma are scarce, we performed a next-generation sequencing that revealed the presence of a hitherto unknown *NTRK2* fusion.

**Interventions:**

Treatment with the TRK inhibitor larotrectinib was initiated, which induced rapid symptom improvement. However, part of the tumor had to be removed shortly afterwards due to local progression. Molecular testing did not demonstrate any alterations accounting for resistance to larotrectinib, with maintenance of the *NTRK2* fusion.

**Outcomes:**

Three months later, imaging confirmed mixed response. While the reason for this remains unknown, the patient is in good condition and continues to receive larotrectinib.

**Conclusion:**

It remains unclear why our patient showed mixed response to larotrectinib and further studies are needed to explore other possible mechanisms of resistance.

## Abstract 2

Supplemental Digital Content is available in the text

## 1 Introduction

Carcinoma ex pleomorphic adenoma (CXPA) is a rare tumor of the salivary glands and is histologically defined as an epithelial malignancy associated with a primary or recurrent benign pleomorphic adenoma. Prognosis is generally poor, with 5-year overall survival ranging from 30% to 50%.^[1]^

Due to the rarity of CXPA, no standardized treatment recommendations are available for the metastatic setting. Various chemotherapeutic regimens and androgen deprivation therapies have shown some benefits as palliative treatments, but generally based on data derived from series with few patients. Targeted therapies to date have not shown any relevant antitumor activity.^[2,3]^ To the best of our knowledge, no case has been published to date that describes treatment of CXPA harboring a neurotrophic receptor tyrosine kinase (*NTRK) 2* fusion using a small molecule inhibitor that targets the tropomyosin receptor kinase (TRK) proteins.

Larotrectinib is an orally available, first-in-class, highly specific TRK inhibitor. The 3 TRKs TRKA, TRKB, and TRKC are encoded by the *NTRK* genes (*NTRK1*, *NTRK2*, *NTRK3*). Oncogenic translocations of these genes produce fusions that link the NTRK kinase domain to the transcriptional regulatory elements and upstream coding regions of many different genes. These lead to constitutive TRK activity that drives oncogenesis.^[4]^ Larotrectinib was approved by the U.S. Food and Drug Administration based on a primary analysis set of 55 patients who achieved a mean response rate of 75% with larotrectinib treatment.^[5,6]^ More recently, it was also approved by the European Medicine Agency for treatment of patients with advanced *TRK*-fusion–positive solid tumors.

## 2 Case presentation

A 63-year-old male journalist was diagnosed with CXPA of the left parotid and parapharyngeal space. He underwent tumor excision, but after 1.5 years, he experienced parapharyngeal recurrence. The tumor was again resected, and local brachytherapy was administered (32 Gy). Over the next 5 years, 3 more surgical interventions followed because of local recurrence in the left parotid, parapharyngeal space and/ or the left ear, until further operations were not feasible, and systemic therapy was required.

As data on the efficacy of systemic therapies for inoperable CXPA are scarce,^[2]^ we performed molecular and immunohistochemical characterization of the tumor tissue. Next-generation sequencing (NGS) using the Oncomine^TM^ Focus Assay Panel showed no relevant mutations, although immunohistochemistry revealed high androgen receptor expression (80%). Androgen deprivation therapy was discussed with the patient, but he rejected this and requested a watch-and-wait strategy.

Within 1 year, the patient developed increasing swelling and pain around the left ear and numbness of the left half of the face. Magnetic resonance imaging and positron emission tomography/computed tomography scans showed tumor progression in the left parotid, below the left ear, and in the parapharyngeal space, as well as metastases of the lungs and cervical lymph nodes.

Androgen deprivation therapy was still not an option according to the patient wishes. Another NGS assessment that focused particularly on *NTRK* fusions (Archer FusionFlex Panel) was performed based on his archival tumor tissue. This revealed an *NTRK2* fusion (*ZCCHC7* [Exon 2]/ *NTRK2* [Exon 16]), which to the best of our knowledge has not been described to date. These fusions can be targeted with TRK inhibitors, such as larotrectinib.^[4]^ After the patient had given consent, treatment was initiated with oral larotrectinib 100 mg twice daily.

The symptoms of the patient regressed within the first 2 weeks of treatment. He discontinued his pain medication, and the numbness of his face was regressing. However, after one month, the swelling around his ear had increased again.

Positron emission tomography/computed tomography assessment conducted 2 months after the initiation of therapy showed stable disease, although magnetic resonance imaging confirmed a mixed response with a growing mass around the left ear. The locally progressive part of the tumor was removed surgically and was analyzed for clonal loss of the *ZCCHC7*-*NTRK2* fusion gene and other alterations that might explain the resistance to larotrectinib treatment. However, NGS (Oncomine^TM^ Comprehensive Assay v3/Archer FusionPlex Salv GlandDx Panel) performed on the fresh tumor tissue still showed the known *NTRK2* fusion, no mutation in the kinase domain, and no other alterations, particularly for genes in the mitogen-activated protein kinase pathway (Table 1). Meanwhile, larotrectinib treatment was suspended to avoid complications during surgery. The treatment was then resumed after 30 days, and tolerance has been good to date.

**Table 1:** Genes analyzed using the Oncomine^TM^ Comprehensive Assay v3, Archer FusionPlex SalvGlandDx Panel, and Archer FusionPlex Panel.

Analyzed genes												
Hotspot genes				Full-length genes			Copy number genes		Gene fusions inter- and intragenic			
*AKT1*	*ESR1*	*KIT*	*PDGFRA*	*ARID1A*	*FBXW7*	*PTEN*	*AKT1*	*FGFR4*	*AKT2*	*EWSR1*	*MYB*	*PRKD3*
*AKT2*	*EZH2*	*KNS*	*PDGFRB*	*ATM*	*MLH1*	*RAD50*	*AKT2*	*FLT3*	*ALK*	*FGFR1*	*MYBL1*	*PTEN*
*AKT3*	*FGFR1*	*TRN*	*PIK3CB*	*ATR*	*MRE11*	*RAD51*	*AKT3*	*IGF1R*	*ARAXL*	*FGFR2*	*NCOA2*	*PPARG*
*ALK*	*FGFR2*	*KRAS*	*PIK3CA*	*ATRX*	*MSH6*	*RAD51B*	*ALK*	*KIT*	*BCOR*	*FGFR3*	*NF1*	*RAD51*
*ARA*	*FGFR3*	*MAGOH*	*PPP2R1A*	*BAP1*	*MSH2*	*RAD51C*	*AXLA*	*KRAS*	*BRCA1*	*FGR*	*NOTCH1*	*BRAF1*
*AXL*	*FGFR4*	*MAP2K1*	*PTPN11*	*BRCA1*	*NBN*	*RAD51*	*RBRAF*	*MDM2*	*BRCA2*	*FLT3*	*NOTCH4*	*RAF1*
*BRAF*	*FLT3*	*MAP2K2*	*RAC1*	*BRCA2*	*NF1*	*DRNF43*	*CCND1*	*MDM4*	*BRAF*	*FN1*	*NR4A3*	*RB1*
*BTK*	*FOXL2*	*MAP2K4*	*RAF1*	*CDK12*	*NF2*	*RB1*	*CCND2*	*MET*	*CAMTA1*	*FOS*	*NRG1*	*RELA*
*CBL*	*GATA2*	*MAPK1*	*RET*	*CDKN1B*	*NOTCH1*	*SETD2*	*CCND3*	*MYC*	*CCNB3*	*FOSB*	*NTRK1*	*RET*
*CCND1*	*GNA11*	*MAX*	*RHEB*	*CDKN2A*	*NOTCH2*	*SLX4*	*CCNE1*	*MYCL*	*CDKN2A*	*FOXO1*	*NTRK2*	*ROS1*
*CDK4*	*GNAQ*	*MDM4*	*RHOA*	*CDKN2B*	*NOTCH3*	*SMARCA4*	*CDK2*	*MYCN*	*CIC*	*FUS*	*NTRK3*	*RSPO2*
*RAF*	*GNAS*	*MED12*	*ROS1*	*CHEK1*	*PALB2*	*SMARCB1*	*CDK4*	*NTRK1*	*CSF1*	*GLI1*	*NUTM1*	*RSPO3*
*CDK6*	*H3F3A*	*MET*	*SF3B1*	*CREBBP*	*PIK3R1*	*STK11*	*CDK6*	*NTRK2*	*EGFR*	*HMGA2*	*PDGFB*	*SFR*
*CHEK2*	*HIST1H3B*	*MTOR*	*SMAD4*	*FANCA*	*PMS2*	*TP53*	*EGFR*	*NTRK3*	*EPC1*	*JAK2*	*PDGFRA*	*SS18*
*CSF1R*	*HNF1A*	*MYC*	*SMO*	*FANCD2*	*POLE*	*TSC1*	*ERBB2*	*PDGFRA*	*ERBB2*	*JAZF1*	*PDGFRB*	*STAT6*
*CTNNB1*	*HRAS*	*MYCN*	*SPOP*	*FANCI*	*PTCH1*	*TSC2*	*ESR1*	*PDGFRB*	*ERBB4*	*KRAS*	*PHF1*	*TAF15*
*DDR2*	*IDH1*	*MYD88*	*SRC*				*FGF19*	*PIK3CB*	*ERG*	*MAML2*	*PIK3CA*	*TCF12*
*EGFR*	*IDH2*	*NFE2L2*	*STAT3*				*FGF3*	*PIK3CA*	*ESR1*	*MDM4*	*PLAG1*	*TERTT*
*ERBB2*	*JAK1*	*NRAS*	*TERT*				*FGFR1*	*PPARG*	*ETV1*	*MEAF6*	*PRKACA*	*TFE3*
*ERBB3*	*JAK2*	*NTRK1*	*TOP1*				*FGFR2*	*RICTOR*	*ETV4*	*MET*	*PRKACB*	*TFG*
*ERBB4*	*JAK3*	*NTRK2*	*U2AF1*				*FGFR3*	*TERT*	*ETV5*	*MGEA5*	*PRKD1*	*USP6*
*ERCC2*	*KDR*	*NTRK3* *PDGFRA*	*XPO1*						*ETV6*	*MLK2* *MSANTD3*	*PRKD2*	*YWHAE*

A positron emission tomography/computed tomography scan conducted 3 months later revealed an overall stable situation (i.e., progression of the cervical lymph node metastases, but stable disease in the remaining tumor sites), and therefore the larotrectinib treatment continues.

## 3 Discussion and conclusions

Therapy with larotrectinib initially led to rapid regression of the symptoms of the patient caused by metastatic CXPA of the parotid that harbored an *NTRK2* fusion. However, a mixed tumor response was observed clinically after only 1 month, which was confirmed radiologically. The site of focal progression was resected. NGS performed on this tissue detected no changes in the molecular signature that might account for resistance to larotrectinib.

This case thus raises various questions regarding the possible reasons for the mixed tumor response, including tumor heterogeneity, resistance mechanisms, and the therapeutic significance of the hitherto unknown *NTRK2* fusion (*ZCCHC7* [Exon 2]/ *NTRK2* [Exon 16]). Heterogeneity is an important mechanism of resistance to tumor therapy and can be caused by genomic instability and clonal evolution/selection.^[7]^

Cancers generally become more heterogeneous over time, which can result in regionally or temporally diverse collections of cells that have distinct molecular signatures, and thus variable sensitivity to treatment. We looked for such heterogeneity in the locally progressive part of the tumor, but NGS did not reveal any changes in the molecular signature, and in particular, the *NTRK2* fusion remained.

*NTRK* fusions are characteristic of rare cancer types and various pediatric cancers. Only a few common cancers in adults carry *NTRK* fusions, which include those of the salivary glands.^[8,9]^ Across the *NTRK* genes, *NTRK2* is infrequently involved in *TRK* fusions (3%), whereas *NTRK3* is most frequently affected (55%), followed by *NTRK1* (40%).^[6,9]^ However, a recent pooled analysis by Hong et al (2020) revealed efficacy of larotrectinib independent of the *NTRK* gene involved, with objective responses in 79% of patients with TRK-fusion–positive solid tumors.^[6]^ Also, in a large cohort of 11,502 solid malignancies, 31 had *NTRK* fusions, where the most common were *ETV6*:*NTRK3* (n = 10) and *TPM3*: *NTRK1* (n = 6).^[8]^

To the best of our knowledge, the *NTRK2* fusion of our patient (*ZCCHC7* [Exon 2]/ *NTRK2* [Exon 16]) has never been described before, which thus also applies to the efficacy of larotrectinib treatment in this setting.^[8]^ Furthermore, different mechanisms of resistance to larotrectinib have been described. The reasons for primary resistance have not been well explained, and it remains unclear if simultaneous presence of other molecular alterations can influence the efficacy of larotrectinib. *NTRK* fusions are usually mutually exclusive, but rare co-occurrences of the biomarkers *ALK*, *BRAF*, *ERBB2*, *EGFR*, *ROS1,* and *KRAS* have been reported recently.^[9,10]^

Acquired resistance arises from secondary mutations within the ATP binding pocket of the TRK kinase domain, which can include solvent-front substitutions, gatekeeper mutations, and xDFG-motif substitutions in the activation loop. Further “by-pass” resistance can develop; that is, genomic alterations of other receptor tyrosine kinases or downstream pathway mediators. Specifically, *MET* amplification, *BRAF*V600E mutation and hotspot mutations involving *KRAS* have been shown in tumor and/or plasma samples from patients that have shown disease progression on TRK inhibitors.^[4,5]^ In our patient, the short duration of treatment suggests primary rather than acquired resistance. However, the patient had no other oncogenic mutations or alterations, such as those described above.

In conclusion, it remains unclear why our patient showed mixed response to larotrectinib, and further studies are needed to explore other possible mechanisms of resistance. Supplemental Digital Content: "Case V Timeline": http://links.lww.com/MD/F604.

## Acknowledgments

The authors thank Bayer Healthcare for providing the medication as part of an early access program and for the medical review of the manuscript (without influencing the content). A special thanks goes to Bayer (Switzerland) AG for financing the editorial support by J. Moser and the journal submission fees.

## Author contributions

HB, MB and TW followed the patient. TW performed the molecular advising. MH provided pathological support. MP, DH, UP and TW wrote the paper. All of the authors have reviewed and approved the final manuscript.

**Conceptualization:** Thomas Winder.

**Methodology:** Milo Horcic.

**Supervision:** Thomas Winder.

**Writing – original draft:** Magdalena Pircher.

**Writing – review & editing:** Hans Rudolf Briner, Marco Bonomo, Ulf Petrausch, Daniel Helbling, Thomas Winder.

## Abbreviations

Abbreviations: CXPA = carcinoma ex pleomorphic adenoma, NGS = next-generation sequencing, NTRK = neurotrophic receptor tyrosine kinase, TRK = tropomyosin receptor kinase.
